# Uncertainty quantification in ToxCast high throughput screening

**DOI:** 10.1371/journal.pone.0196963

**Published:** 2018-07-25

**Authors:** Eric D. Watt, Richard S. Judson

**Affiliations:** 1 U.S. Environmental Protection Agency, National Center for Computational Toxicology, Research Triangle Park, North Carolina, United States of America; 2 Oak Ridge Institute for Science Education Postdoctoral Fellow, Oak Ridge, Tennessee, United States of America; University of Louisville School of Medicine, UNITED STATES

## Abstract

High throughput screening (HTS) projects like the U.S. Environmental Protection Agency's ToxCast program are required to address the large and rapidly increasing number of chemicals for which we have little to no toxicity measurements. Concentration-response parameters such as potency and efficacy are extracted from HTS data using nonlinear regression, and models and analyses built from these parameters are used to predict *in vivo* and *in vitro* toxicity of thousands of chemicals. How these predictions are impacted by uncertainties that stem from parameter estimation and propagated through the models and analyses has not been well explored. While data size and complexity makes uncertainty quantification computationally expensive for HTS datasets, continued advancements in computational resources have allowed these computational challenges to be met. This study uses nonparametric bootstrap resampling to calculate uncertainties in concentration-response parameters from a variety of HTS assays. Using the ToxCast estrogen receptor model for bioactivity as a case study, we highlight how these uncertainties can be propagated through models to quantify the uncertainty in model outputs. Uncertainty quantification in model outputs is used to identify potential false positives and false negatives and to determine the distribution of model values around semi-arbitrary activity cutoffs, increasing confidence in model predictions. At the individual chemical-assay level, curves with high variability are flagged for manual inspection or retesting, focusing subject-matter-expert time on results that need further input. This work improves the confidence of predictions made using HTS data, increasing the ability to use this data in risk assessment.

## Introduction

The U.S. Environmental Protection Agency (EPA) Toxic Substances Control Act (TSCA) inventory currently lists about 85,000 chemical substances manufactured, processed, or imported in the United States, and roughly 400 new chemicals are added every year [1]. Expensive and lengthy animal-based toxicology studies are not able to keep pace with this large inventory of chemicals. For those few chemicals where there is in vivo data, extrapolation across species, doses, and life stages is hindered by a lack of mechanistic information.

These limitations represent a need to supplement traditional animal toxicity studies. The National Research Council (NRC) outlined a long-term vision for including new in vitro studies to complement, extend, and, where applicable, replace animal studies [2]. The stated goals of this approach included lowering costs, decreasing animal use, increasing throughput, providing coverage of mechanism and pathways, and increasing the human relevancy of toxicity results. The EPA has pursued these objectives through the ToxCast program [3,4] as well as through participation in the Toxicology in the 21st Century (Tox21) program, an interagency collaboration among the EPA, National Institutes of Health's National Center for Advancing Translational Sciences (NIH's NCATS), the National Toxicology Program (NTP), and the Food and Drug Administration (FDA) [5,6].

Together the ToxCast and Tox21 programs have had a transformative impact on how chemicals are evaluated for safety and hazard towards effects on both human health and the environment. Current chemical coverage represents ~2000 chemicals studied in >800 assays representing ~400 biological targets and pathways, and an even larger set of >8000 chemicals have been tested in a subset of these assays [7–9]. Assay sources include: cell-free binding displacement and enzymatic reactions with radioactive, colorimetric, and/or fluorescence detection (Novascreen/NVS) [10,11]; in cell protein-fragment complementation assays with fluorescence detection (Odyssey Thera/OT) [12,13]; in cell multiplexed reporter transcription unit assays with RNA transcript level detection (Attagene/ATG) [14]; cell proliferation monitored by real-time electronic sensing (ACEA) [15]; high-content multiparameter quantitative digital imaging (Vala) [16]; embryonic stem cell differentiation and cytotoxicity (NHEERL MESC) [17,18]; zebrafish developmental disruption (NHEERL Zebrafish) [19–21]; stress response and nuclear receptor signaling (NCATS/NCGC/Tox21) [22–27]; high content imaging of HepG2 cells (Apredica/APR) [28]; human primary cell protein expression (BioSeek/BSK) [29]; and newly developed assays within the EPA (NCCT TPO). [30]

The rich mechanistic information provided by such a large and diverse dataset has lead to the results being used in many different contexts. Predictive models have been developed for reproductive toxicity [14], hepatotoxicity [31,32], carcinogenicity [33], developmental toxicity [34], vascular development toxicity [35,36], and estrogen receptor (ER) disruption [37,38]. In addition, researchers have used the large amount of data in HTS to build computational models to predict HTS results for untested chemicals where little is known about their toxicity [39,40]. Adverse outcome pathways (AOPs) [41,42] and tools like the Toxicological Prioritization Index (ToxPI) [43,44] leverage the unique mechanistic detail provided by ToxCast in vitro studies and provide a means of connecting ToxCast and Tox21 HTS data to endpoints meaningful for risk assessment. With this information, results from ToxCast have been used for prioritizing chemicals for more targeted testing [45]. The ability to link HTS results to high throughput exposure estimates [46] and in vivo assays using in vitro to in vivo extrapolation (IVIVE) pharmacokinetics measurements [47–49] has allowed HTS results to be increasingly used in risk assessment [5,50,51].

However, there have been studies highlighting limitations to predictivity from HTS results [52,53]. While numerous factors can contribute to reduced predictivity, the uncertainty in concentration-response parameters of the HTS data has to date been an underexplored contributor. While the need for incorporating quantitative uncertainty analysis for high throughput screening has been acknowledged, the increased computational expense has limited the application of robust statistical methods [54–56].

There are several challenges for calculating uncertainty in HTS data. The choice of a method to quantify uncertainty must consider these issues.

1. Diverse use. There are multiple ways that HTS values are incorporated into downstream analysis. Past studies have made use of: binary activity calls [57]; individual fitted parameters such as potency [34]; or all fit parameters [37,38].
2. Diverse assay space. The different assay sources, technologies, and techniques are of great benefit when building models and identifying technology confounders, but this diversity can complicate calculations of uncertainty. For example, a given chemical may be tested with nine concentrations and a single replicate in the ATG assays (n = 9 observations), four concentrations in triplicate in the OT assays (n = 12), and 15 concentrations in triplicate in the Tox21 assays (n = 45). The response in an ATG assay may reach 5-fold induction while NVS and Tox21 report percent inhibition that can be 100% or greater. An algorithm to estimate uncertainty must work on both the highly sampled Tox21 data as well as the sparsely sampled ATG results.
3. Data size. The October 2015 ToxCast v2 release contained over 2.4 million concentration-response curves while the current internal database has expanded to over 2.7 million. This presents a challenge in both computational time as well as data storage. Previous studies attempted to balance the computational cost with statistical accuracy, employing multistage classification algorithms [58] or asymptotic methods [54]. However, these studies focused on NCATs/Tox21 as a single assay source. While the current method of processing ToxCast data includes an asymptotic approximation of parameter uncertainty, values for small sample sizes may be inaccurate and uncertainty estimates for parameters near constraint boundaries will be undefined.
4. Diverse users. The method should be of utility to a researcher processing a new assay, a student building a model on HTS results, or a scientist performing risk assessment for a policy decision. An overly complicated method that requires user input and subject-matter-expert tuning would limit the applicability of the uncertainty results.

In this paper, we introduce non-parametric bootstrap resampling [59,60] as a method that can calculate uncertainty estimates in HTS data. While the computational expense of a large number of resamples has hindered the adoption of bootstrap methods in the past [56,61], advancements in computational power have made the method feasible to apply to the ToxCast HTS dataset. We describe a bootstrap implementation suitable for incorporation in the ToxCast pipeline and explore how the method meets the challenges for quantifying uncertainty in a diverse dataset like ToxCast.

As a case study, we explore an application to the ToxCast estrogen receptor (ER) model for bioactivity [37,38]. Calculating uncertainty in this model must meet all of the challenges described above. The model calculates area under the curve (AUC) values for a given chemical using the fitted curves for that chemical from 18 ER assays. Uncertainty in the fitted curve requires that we capture uncertainty in the hit call, model selection, and all fit parameters from the winning model (challenge 1). The assays in the model include ACEA, ATG, NVS, OT, and Tox21 assays, representing many of the assay sources, technologies, and concentration sampling schemes found in the ToxCast library (challenge 2). With 18 assays and ~1800 chemicals, ~30k concentration-response curves must be bootstrapped to run the complete model (challenge 3). This model is well characterized and has recently been approved to replace in vivo tests as part of the Endocrine Disruptor Screening Program (EDSP) Tier 1 battery [62,63]. This means that not only do developers need to understand the uncertainty in the model prediction, but the method used must be easy to communicate to regulators and industry partners who make use of the model as part of their risk assessments (challenge 4).

## Results and discussion

### Bootstrap selection and smoothing parameter

While numerous bootstrapping algorithms have been described in the literature [63–66], we chose to use smoothed nonparametric resampling (smooth bootstrap). There are minimal assumptions used in this method. First, the observed response values are physically possible (a small assumption since they were observed). Second, for each response value there is some noise and uncertainty included in the measurement. While non-smoothed nonparametric resampling (case bootstrap) removes the second assumption, this comes at the cost of jagged parameter distributions in samples with few points and the inability to bootstrap curves with only a single biological replicate. Smoothing removes the jaggedness, slightly increases the amount of variation, and allows resampling for curves with only a single biological replicate. Because the nonparametric methods do not rely on a specific functional form of the curve, they can be used to quantify the uncertainty in model selection and activity call as well.

Methods that resample residuals make a hard assumption on the model. Since the residuals are calculated from the fitted curve, the choice of function must be made prior to bootstrapping, removing the ability to capture uncertainty in model selection and activity. Directly resampling the residuals makes an additional assumption that the variance of errors is constant, and like case resampling, this method can result in jagged distributions for curves with few points. Wild resampling removes the assumption of homoscedasticity [63–66], and depending on the random variable used to multiply the residuals, can smooth out some of the jaggedness in residual resampling. However, the choice of random variable is not trivial and may need to be adjusted for different assay types. The wild bootstrap is also sensitive to the regression method and the pattern of heteroscedasticity [66].

Based on the comparisons summarized in Table 1, the smooth bootstrap was selected as most applicable to the diverse datasets found in ToxCast in general and the ER assays in particular. The amount of noise added into the smooth bootstrap can have a significant impact on the results. Not enough and the results will be much like case resampling: often discrete bins of parameter values will be observed for curves with few points. If the random noise is too high, the uncertainty calculated will be artificially inflated. Fortunately, the ToxCast pipeline already contains an estimate on the noise. In the data fitting process, the baseline median absolute deviation (bmad) is calculated by binning the response values of the lowest two concentrations for every chemical, and then computing the scaled mad, $bmad=1.4826*median(|X_{i}-\tilde{X}|)$ where X_i_ is the i^th^ value in the binned baseline response values and $\tilde{X}$ is the median of the baseline response values [67,68]. We use the median and mad rather than mean and standard deviation because a small number of chemicals are highly potent and have a response even at the lowest concentrations. Within the ToxCast pipeline, the bmad is used as a measure of noise. Curves without a median response at any concentration greater than 3 * bmad are not fit to the hill or gnls models. In addition, many assays have the cutoff value for a statistically significant response set to a multiple of the bmad, with 3, 6, and 10x bmad frequently used.

**Table 1:** Applicability of bootstrap methods to assays with only one measurement per concentration, determination of a winning model, and calculating a hit call probability.

	Case	Smooth	Residuals	Wild
**Sample Single Replicate**		y	y	y
**Model Selection**	y	y		
**Hit Call Percent**	y	y		

Given that the assumption that bmad represents the noise in assay data is already built into how the ToxCast pipeline is constructed, maintaining that assumption for the smooth bootstrap makes sense. Therefore, we sampled from random noise calculated from a normal distribution with standard deviation equal to the bmad for that assay.

**Fig 1:**
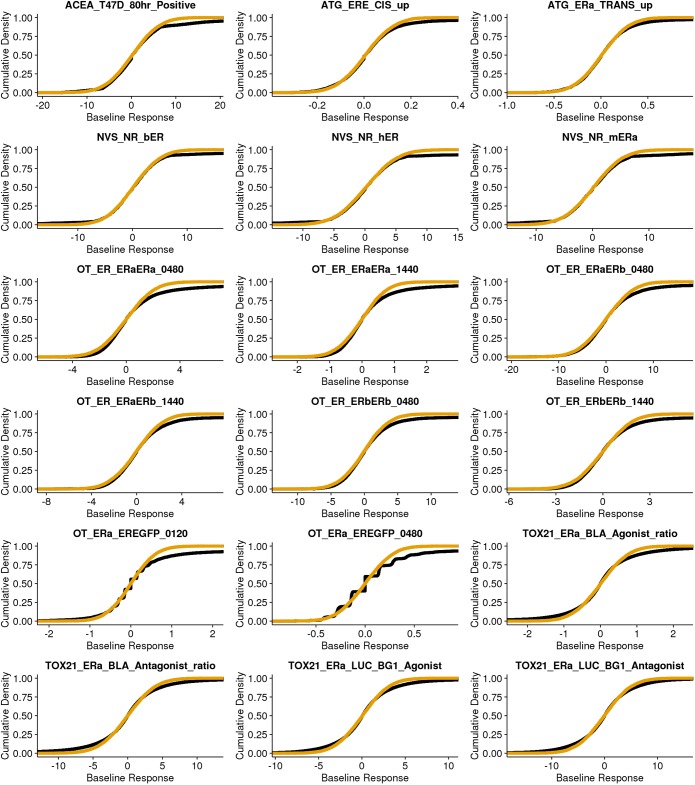
Comparison of normal distribution with standard deviation equal to bmad (orange line) and the empirical cumulative distribution function (ecdf) for points used to calculated bmad (black line). For each assay, the bmad is calculated as the scaled mad of the response values for the lowest two concentrations per chemical. Deviations between the ecdf and the normal distribution at higher response values can be attributed to highly potent chemicals with a biological response at the lowest two concentrations as well as sources of noise that are from a non-normally distributed process.

We compare the empirical baseline values for the two lowest concentrations tested across all chemicals to the normal distribution built on the bmad in Fig 1 for all 18 ER assays. In each pane,l the empirical values are plotted as the empirical cumulative distribution function in black, while the normal distribution with standard deviation set to the bmad is plotted as orange lines. In all 18 assays, there is substantial similarity between the two distributions. This indicates that the normal distribution is a good approximation to the actual underlying distribution.

It is also clear that the largest deviation occurs as the response value increases. This occurs due to highly potent chemicals with activity within the lowest two concentrations of the tested range. The effect is greatest in the Odyssey Thera assays (OT_ER). Because these assays were tested at fewer concentrations clustered at the higher concentration values, more chemicals show activity in the baseline. In contrast, the Tox21 assays were tested at more than 100-fold lower concentration. Because of this, there is a much smaller deviation between the two distributions for the Tox21 assays. In all cases, the normal function makes an excellent approximation for the background noise in the assay, highlighting that a normal distribution built on the bmad represents a good choice for sampling noise in the smooth bootstrap as well as providing confidence in the use of bmad within the pipeline for hit call cutoffs.

### Confidence intervals in model parameters

**Fig 2:**
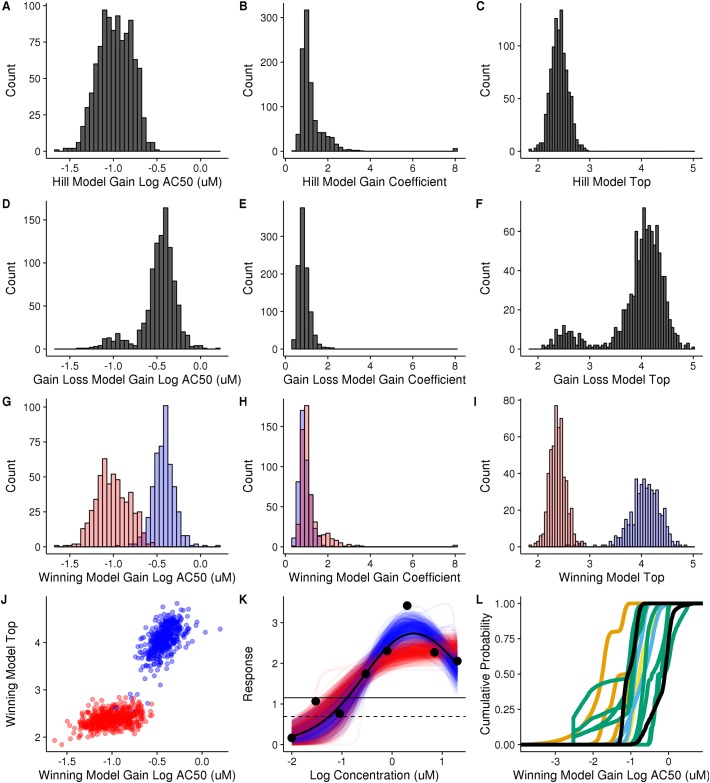
Analysis of bootstrap distribution of model parameters for bisphenol AF in ATG_ERa_TRANS_up assay. **A-C**: Values for the hill model log(AC50) (A), coefficient (B), and top (C) parameters for all 1000 bootstrap samples. **D-F**: Values for the gain loss model gain log(AC50) (D), gain coefficient (E), and top (F) for all 1000 bootstrap samples. **G-I**: Values for the winning model gain log(AC50) (G), gain coefficient (H), and top (I) for all 1000 bootstrap samples, colored by winning model (hill = red, gain loss = blue). **J**: Correlation plot of winning model top vs. winning model gain log(AC50), colored by winning model (hill = red, gain loss = blue). **K**: Normalized experimentally measured values (black circles) and winning model (gain loss, black curve). Subset of fitted bootstrap resamples, with winning hill (red lines) and gain loss (blue lines) models plotted. Horizontal black lines represent 3x bmad (dashed) and activity cutoff (solid). **L**: Comparison to results from other assays. Cumulative empirical distribution function of winning model gain log(AC50) value for all bisphenol AF samples in all assays where the experiment results were determined to be a positive hit. Curves are colored by assay source, with TOX21 black, NVS orange, ATG sky blue, OT bluish green, and ACEA yellow.

The most straightforward analysis of the bootstrap results is to consider the distribution of the model fit parameters. The three parameters fit in the hill and gain loss (gnls) models are the log(AC50), top, and hill coefficient. For each parameter, we calculate the distribution of values using the bootstrapping method, and can then calculate the 95% confidence interval by taking the 0.025 and 0.975 quantiles of the sample values. An example using bisphenol AF in the Attagene ERa TRANS assay is shown in Fig 2.

It is clear that the hill (Fig 2A–2C) and gnls (Fig 2D–2F) parameters are not always normally distributed. The hill log(AC50) (A), hill top (C), and gnls coefficient (E) are roughly Gaussian. However, the other parameters have different distributions. A long tail is observed for the hill coefficient (B). The gnls log(AC50) (D) has large tails on both sides of the distribution and gnls top (F) is bi-modal. This indicates that a simple normal distribution and associated confidence intervals cannot be assumed to be applicable.

### Model selection and hit determination

While the distribution of an individual model parameter is informative, many analyses of ToxCast data make use of the winning model rather than focusing on the hill or gnls models specifically. In addition to individual model fit parameters, each bootstrap sample has the calculated Akaike information criteria (AIC) for all three models. Using this, we choose a winning model for each resampled curve by selecting the lowest AIC using the same algorithm used in the ToxCast pipeline point estimate. Fig 2 is an example where the winning model can vary between bootstrap samples. While there are 1000 measurements for the hill log(AC50) and the gnls log(AC50), not all of those represent curves where those models are the winning models. For each bootstrap sample, we select the log(AC50) that corresponds to the winning model and pool those results (Fig 2G). Comparing Fig 2A, 2D and 2G, it is clear that distribution of the winning model log(AC50) is broader than either the hill or gnls log(AC50) and is bimodal, representing the combination of the two different distributions from the hill and gnls subsets. Fig 2H and 2I highlight the winning model gain coefficient and top parameter distributions, respectively. The uncertainty in the winning model is adding to the uncertainty in the potency parameter.

By keeping the parameters paired with the bootstrap sample, the correlations between parameters can be explored. In Fig 2J, the log(AC50) and top parameters for the winning model in all 1000 bootstrap samples are shown. Notably, the hill and gnls components of the winning model parameters have different correlations. The shape and angle are different, with a stronger correlation between the log(AC50) and the top parameters observed in the gnls than in the hill model.

The reason for the shift in efficacy and potency between the two models is clarified by examining the bootstrap sample curves (Fig 2K). The response at 0.3 log(uM) is 3.4, more than one unit greater than the preceding and following concentrations. In the ToxCast pipeline, this data fits to the gnls model (solid black curve). When bootstrapped, however, uncertainty in the points shifts the winning model, such that out of 1000 bootstrap samples the hill (red) and gnls (blue) models are the chosen 526 and 474 times, respectively. While the maximum of the blue and red curves differs slightly, ~0.5 response units, the top parameter for the gnls and hill equations represents the asymptotic value for the gain direction only. In the gnls model, this is clustered around 4, much greater than the 2.5 to 3 value represented by the maximum of the gnls curve or the ~2.5 clustering of the hill model (Fig 2I). Because the log(AC50) represents the calculated concentration where the response is half the value of the top parameter, the shift in the top between gnls and hill manifests as a shift in the log(AC50) as well.

Finally, we can make a hit call for each sample using the same algorithm as the pipeline: set each bootstrap sample to a hit if the winning model is hill or gnls, the max median is > cutoff, and the winning model top is > cutoff. As the winning model can vary between bootstrap samples, the hit call can change as well. While the current ToxCast pipeline provides a binary Yes/No hit call determination, bootstrapping provides a means to calculate a hit probability. For example, if 500 of the 1000 bootstrap samples failed to meet the hit call criteria, the hit percent would be only 50%, suggesting lowered confidence in the hit call for that curve.

**Fig 3:**
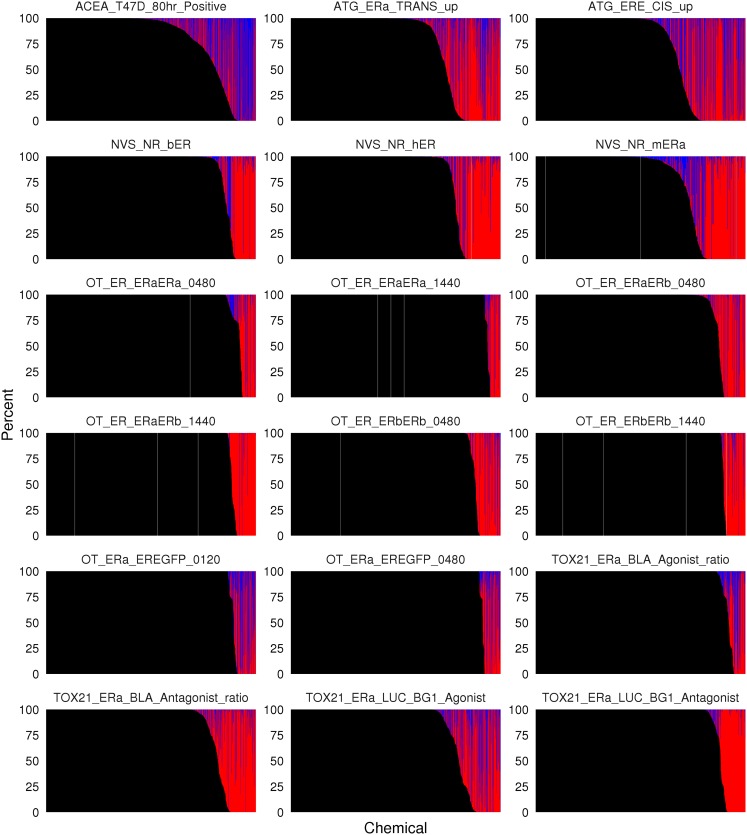
Model selection in hit call probability for sixteen estrogen receptor agonist assays. For each plot, chemicals are ordered on the x-axis based on their hit call probability. The y axis indicates the percent of bootstrap resamples that were calculated to be a positive hit with a hill model (red), gain loss (blue), or a negative hit (black).

We explored the uncertainty in the hit call and model selection for all 1811 chemicals in 18 ER assays in the ToxCast database. For each chemical assay pair, a model selection and hit call was made for each bootstrap sample. Therefore, for each curve a hit probability was calculated, and among the samples that were hits the ratio of hill to gnls was determined. These results are shown in Fig 3.

The percentage of chemicals with a hit probability greater than 0 but less than 1 varies substantially between assays. In ACEA_T47D_80hr_Positive over 25% of the curves have a hit probability between 0 and 1. In contrast, many of the Odyssey Thera assays have a much smaller number of chemicals in this probability range. The steepness of the transition from 0% to 100% hit call is driven by the noise of the assay, the choice of cutoff, and the range of responses observed. The Odyssey Thera assay results have a sharper transition from 0% to 100% than is observed in the ACEA dataset, across the same set of 1811 chemicals tested.

Propagation of model parameters, model selection, and hit call probability will vary depending on the final use case. If the assay hit call is an input into a model, such as building a QSAR model to predict assay activity, one option is to leave out any chemical with a hit probability between 0 and 1. Another approach would be assign a hit probability threshold for a chemical to be included as a positive or negative (e.g. >0.75 or <0.25). In this study, we explore applications to the ER Model, which is handled differently.

### Application to estrogen receptor model

The ToxCast ER model for bioactivity calculates AUC values for a given chemical using the fitted curves for that chemical from the 18 ER assays. The model returns an AUC value for 26 different "receptors" in the pathway model corresponding to predicted patterns of activity. These include agonist, antagonist, and pseudoreceptors (technology-specific assay interference activity). The model AUC values are scaled so that chemicals with no ER activity have an AUC value of 0 and the positive agonist control 17*α*-Ethinylestradiol used in all assays has an AUC(agonist) value of 1 [37]. A cutoff of 0.1 AUC(agonist), corresponding to assay potency of ~100 uM, was set for a chemical to be considered positive while scores 0.001 < AUC < 0.1 were considered inconclusive [38].

Calculating the uncertainty in the ER AUC value requires meeting the four challenges highlighted in the introduction. The model is built on the entire curve for each chemical-assay pair, including all fit parameters, model selection, and activity call. Robustness is introduced to the model by using 18 assays from five different sources using different assay technologies. With 1811 chemicals and 18 assays, over 32,000 concentration-response curves are used when calculating the model scores. The model also has diverse applications. In addition to being used for regulatory decisions as part of the EDSP Tier 1 screening battery [62], the model has also been used to build QSAR models so that tens of thousands of additional chemicals can be screened *in silico* for estrogen agonism [39]. Therefore, the ER model makes an ideal use case for understanding how uncertainty quantification can be incorporated into analyzing HTS data. Uncertainty in all of the fit parameters, model selection, and activity call must be propagated for thousands of chemicals and 18 assays, in a way compatible with different assay technologies and giving a result useful for both scientific analysis and regulatory risk assessment.

**Fig 4:**
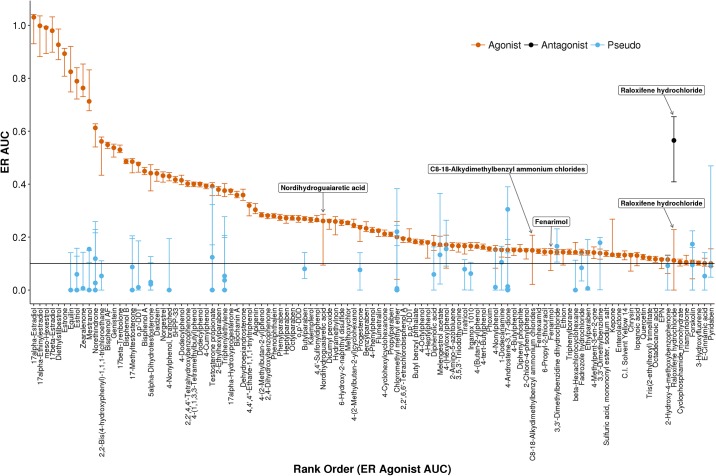
Estrogen receptor model AUC values for chemicals with an AUC(Agonist) value > 0.1. Point estimates for agonist (red), antagonist (black), and pseudoreceptor (blue) values are marked by circles for all AUC values with an upper 95% confidence interval > 0.1. Error bars indicate the 95% confidence interval obtained by bootstrap resampling.

By calculating the ER model score for each bootstrap sample, a distribution of ER model scores was determined. The ER AUC(Agonist), shown in red in Fig 4, is plotted for all chemicals with an AUC(Agonist) value > 0.1. The bootstrapped uncertainty in this value is represented by error bars which mark the 2.5% and 97.5% quantile of the distribution of ER AUC(Agonist) values for that chemical. Similar values and uncertainties are plotted for ER AUC(Antagonist) and AUC(pseudoreceptor) values if the 97.5% quantile of the AUC value is greater than 0.1.

For many chemicals, the uncertainty around the ER AUC(Agonist) value is small. Because the AUC value is calculated by aggregating results from 18 assays, noise from one assay will tend to be averaged out by noise in another assay, providing robustness to the AUC value. Chemicals with AUC(Agonist) values greater than 0.6 tend to have larger error bars. These chemicals are highly potent ER agonist control chemicals and are often active even at the lowest concentration tested in the ToxCast assays (S1 Fig, 10.23645/epacomptox.6062650). If the low response values of the hill curve are not sampled (i.e. the chemical is active at all concentrations) the exact value of the potency is difficult to determine and larger uncertainty in the potency estimate translates into greater uncertainty in the AUC value. Other chemicals, like raloxifene hydrochloride, have a larger uncertainty in the AUC(Agonist) value because there is another AUC value with similar weighting within the model, in this case AUC(Antagonist). The uncertainties around both the agonist and antagonist values are large because each bootstrap sample might skew towards the agonist or antagonist models being dominant (S1 Fig, 10.23645/epacomptox.6062650). Values for AUC(pseudoreceptor) have high uncertainties in general. These values are calculated based on a subset of the assays, and are therefore not as robust as the AUC(Agonist) value.

**Fig 5:**
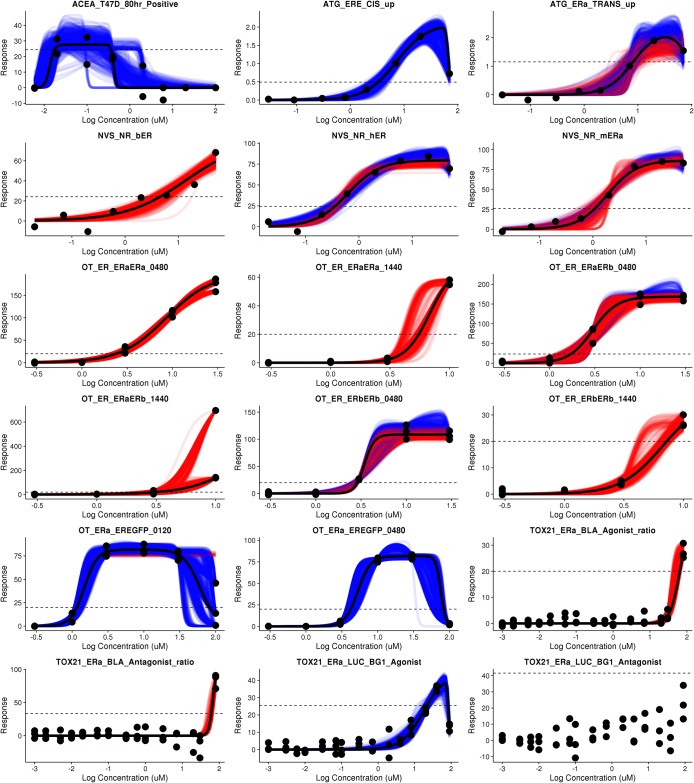
Nordihydroguaiaretic acid bootstrap curves. Each of the 18 ER assays are shown in a separate panel with the assay cutoff indicated with a dashed horizontal line. Circles represent the pipeline normalized concentration-response data and the solid black line indicates the winning model fit to the data if the hit call was positive. TOX21_ERa_LUC_BG1_Antagonist was not a hit in the pipeline therefore no black line is drawn. All bootstrap curves with a positive hit call are drawn with hill and gns models colored red and blue respectively. All assays had a 100% hit call in the bootstrap results except for ACEA_T47D_80hr_Positive where 602 of the 1000 samples had a positive hit call and assays ATG_ERa_TRANS_up and TOX21_ERa_LUC_BG1_Agonist where a single bootstrap replicate in each assay was inactive.

There are, however, a few chemicals that have relatively large AUC(Agonist) uncertainty values. Nordihydroguaiaretic acid, C8-18-Alkydimethylbenzyl ammonium chlorides, and fenarimol are notable in that the 95% CI crosses the 0.1 AUC activity threshold. A closer examination of the first of these, nordihydroguaiaretic acid, is explored in detail in Fig 5. By plotting the bootstrap results for all 18 ER assays for this chemical, the contribution to the ER AUC uncertainty from each assay is explored. Almost all the assays have a relatively narrow range of intra-assay potency values. However, the ACEA_T47D_80hr_Positive data has a significantly more potent AC50 of ~10 nM. Additionally, because the efficacy is barely above the activity cutoff, the bootstrap samples are active only ~60% of the time. This high potency estimate combined with high uncertainty in the activity call translates into large uncertainty in the ER AUC(Agonist) value. For the bootstrap samples where the ACEA data is called active, the high potency drives the ER AUC(Agonist) value up. When the bootstrap samples are inactive, the calculated AUC values decrease. Therefore, we conclude that the large uncertainty in the nordihydroguaiaretic acid ER AUC(Agonist) value is driven primarily by the large uncertainty in the ACEA activity call for this chemical. One follow-up to such a finding would be to rerun the assay driving the overall large uncertainty.

**Fig 6:**
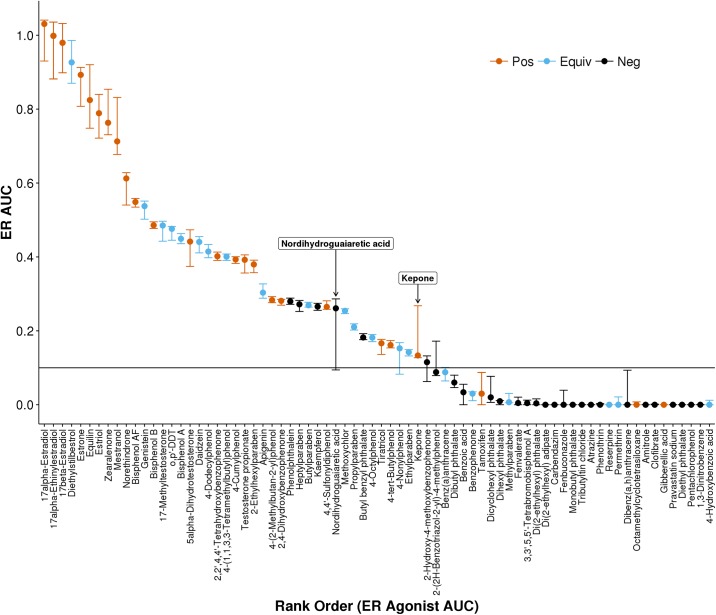
Estrogen receptor model agonist AUC values for chemicals tested at least twice in the uterotrophic assay. Point estimates for agonist are colored by the uterotrophic consensus result being positive (red), equivocal (blue), and negative (black). Equivocal results in the uterotrophic assay indicate some tests were positive while others negative. Error bars indicate the 95% confidence interval obtained by bootstrap resampling.

Because one of the purposes of the ER model is to predict *in vivo* activity, it is informative to compare model scores to known *in vivo* activity for the subset of chemicals that have been tested *in vivo*. In Fig 6 we plot the ER AUC(Agonist) value for all chemicals that have at least two guideline-like studies in the uterotrophic assay, and color the values based on the results of the *in vivo* experiments. The majority of *in vivo* positives are above the 0.1 AUC cutoff and negatives below 0.001. The balanced accuracy of the model is >80% [38] with many of the false positives and false negatives justified biologically (e.g. differences in metabolism or clearance). By adding uncertainty quantification, we are able to further give context around the model score and to increase confidence decision making. The majority of compounds small uncertainty around their model score, and therefore a decision based on that model score can be made for confidently. Others, such as 4-nonylphenol and benz(a)anthracene, have confidence intervals that cross the activity cutoff, and therefore these cannot be confidently predicted to be ER *in vivo* active or not. Similarly, one of the false negatives in the model, tamoxifen, has a relatively large uncertainty that spans into the inconclusive range of model values. By quantifying the uncertainty around the model score, results with low confidence can be flagged to avoid incorrect decision making.

## Conclusions

Using smooth nonparametric bootstrapping, we were able to quantify uncertainty in model fits to the experimental data, and propagate that uncertainty throughout the analysis of the data. Through the use of the ER model, we showed that the method is applicable to the use cases highlighted in the introduction. We calculated the uncertainty in all model fit parameters, and then propagated that uncertainty through model selection, hit call, and finally the ER AUC calculation. This method worked on data from numerous assay sources and technologies, and was fast enough to allow the full propagation to be calculated for all 1811 chemicals. The limited number of assumptions and tuning parameters in applying the method make it simple for non-subject-matter-experts to apply the calculation to other analyses and provides confidence in interpreting the results from the uncertainty quantification. The latter is particularly important for analyses like the ER model that are used in a regulatory context.

One question that might be raised is how our approach compares with the asymptotic, maximum likelihood method for estimating confidence intervals. The estimation process we use includes features that invalidate standard asymptotic theory for evaluating the uncertainty of estimates. First, the parameter space is bounded, and estimates do end up on the boundaries of the space. Standard theory requires estimates to fall on the interior of the parameter space, and are invalid on the boundary. Second, we fit multiple models, and select the model with the best AIC. Again, standard theory does not apply. Finally, we believe that the sample sizes are such that we could not trust the asymptotic theory, even if the two issues above were not true. Thus, we believe that one would want to use some sort of resampling method in any case to more reliably quantify uncertainty.

By quantifying uncertainty in the ER model score, we were able to better understand the semi-arbitrary activity cutoff for *in vivo* ER activity prediction. The distribution of ER model scores gives a measure of confidence around this cutoff. In particular, we were able to identify a false positive by the large uncertainty around the ER AUC(Agonist) value, and then take a closer look at the individual curves used to calculate this value and identify which curve was contributing the most variability. Flagging for closer inspection is a powerful aspect of this uncertainty quantification. With over 32,000 concentration-response curves used to calculate the 1811 ER AUC values, a manual inspection of every curve would be difficult and error prone. By limiting the manual inspection to only those chemicals with large variability and quantifying which curves are contributing to that variability, subject-matter-expert time is optimized for studying only the most difficult examples. As the number of assays, molecular targets, tested chemicals, and analyses grows, tools that target the need for manual inspection increase in importance. Uncertainty quantification is an important component of this analysis pipeline.

## Methods

### Estrogen receptor concentration-response data

Concentration response data used in this study was obtained from 18 ER assays in the ToxCast database. All data was obtained from invitrodb_v2 released October 2015. A summary of the assays used in this study can be found in Table 2.

**Table 2:** Estrogen receptor assays included in this study.

Assay	Source	Normalization	Organism	Tissue	Cell
ACEA_T47D_80hr_Positive	ACEA	percent_activity	human	breast	T47D
ATG_ERE_CIS_up	ATG	log2_fold_induction	human	liver	HepG2
ATG_ERa_TRANS_up	ATG	log2_fold_induction	human	liver	HepG2
NVS_NR_bER	NVS	percent_activity	bovine	uterus	NA
NVS_NR_hER	NVS	percent_activity	human	NA	NA
NVS_NR_mERa	NVS	percent_activity	mouse	NA	NA
OT_ER_ERaERa_0480	OT	percent_activity	human	kidney	HEK293T
OT_ER_ERaERa_1440	OT	percent_activity	human	kidney	HEK293T
OT_ER_ERaERb_0480	OT	percent_activity	human	kidney	HEK293T
OT_ER_ERaERb_1440	OT	percent_activity	human	kidney	HEK293T
OT_ER_ERbERb_0480	OT	percent_activity	human	kidney	HEK293T
OT_ER_ERbERb_1440	OT	percent_activity	human	kidney	HEK293T
OT_ERa_EREGFP_0120	OT	percent_activity	human	cervix	HeLa
OT_ERa_EREGFP_0480	OT	percent_activity	human	cervix	HeLa
TOX21_ERa_BLA_Agonist_ratio	TOX21	percent_activity	human	kidney	HEK293T
TOX21_ERa_BLA_Antagonist_ratio	TOX21	percent_activity	human	kidney	HEK293T
TOX21_ERa_LUC_BG1_Agonist	TOX21	percent_activity	human	ovary	BG1
TOX21_ERa_LUC_BG1_Antagonist	TOX21	percent_activity	human	ovary	BG1

### ToxCast data pipeline

Normalized concentration-response points, model parameter point estimates, and hit call results are included in invitrodb_v2. All model fits to the data used the ToxCast data pipeline R package tcpl version 1.2.2 as described previously [67,68]. The steps relevant to this study are briefly described.

Three models are fit to the normalized concentration-response data using maximum-likeliood to estimate the parameters. Robust estimation was provided by basing the likelihood function on Student’s t distribution with 4 degrees of freedom [69]. The Nelder-Mead algorithm was used to carry out the optimization. All experimental data concentrations *x[i]* and model potency parameters (*ga*, *la*), are expressed as the log10(concentration) where concentration is in uM. The constant ('cnst') model, with constant value of zero response, is given by:

μ[i]=0

The second model fit is the constrained hill ('hill') model:

$$\mu [i]=tp(\frac{1}{1+10^{(ga-x[i])*gw}})$$

subject to constraints:

0≤tp≤1.2×max(resp)

min(conc)-2≤ga≤max(conc)+0.5

0.3≤gw≤8

Fitted parameters are the top asymptote (*tp*), concentration at which the activity is half that of the top asymptote (*ga*), and hill coefficient (*gw*) with constraints indicated. All constraints are subject to the max(resp), min(conc), and max(conc) for the data fit, not at the assay level. The bottom asymptote is set to zero. Notably the constraints on *tp* being greater than zero coupled with the bottom asymptote at zero forces the model to fit only in the gain direction.

The final model fit is the constrained gain loss ('gnls') model. This model is constructed as product of a gain direction hill model and a hill model that operates in the loss direction with shared top and bottom asymptotes:

$$\mu [i]=tp(\frac{1}{1+10^{(ga-x[i])*gw}})(\frac{1}{1+10^{(x[i]-la)*lw}})$$

subject to constraints:

0≤tp≤1.2×max(resp)

min(conc)-2≤ga≤max(conc)

0.3≤gw≤8

min(conc)-2≤la≤max(conc)+2

0.3≤ga≤18

la-ga>0.25

In addition to the previous gain hill parameters, the gnls model adds two loss parameters: the concentration at which the activity of the in loss direction is half that of the top asymptote (*la*) and the loss direction hill coefficient (*lw*). Constraints on these parameters are indicated above.

The fitted models are constrained to model *in vitro* toxicology data to accommodate heterogeneous assay vendors and technologies. For each curve analyzed using the ToxCast Data Pipeline, the constant, constrained Hill, and constrained gain-loss functions are fit, with selection of the winning model corresponding to the minimum Akaike information criterion (AIC)[70]. For simplicity in understanding the shape of the dose-response curve for chemical-induced bioactivity, all data in this version of the ToxCast Data Pipeline have been plotted in the “up” direction; i.e., all curves go in the same direction.

The constraints on the Hill and gain-loss functions are designed to ensure that: (1) an AC50 from the positive portion of the curve is derived for each curve with a positive response, as this is important summary information for toxicological applications; (2) that the AC50 is a conservative estimate of bioactivity, with constraints that allow for estimation of AC50s that may be below the concentration range screened; and, (3) that smoother curves are generated, such that estimated AC50s are not overly conservative for toxicology applications. For the Hill and gain-loss functions, the bottom asymptote is forced to zero. For the Hill function and gain portion of the gain-loss function, the top asymptote is constrained from 0 to 1.2(maximum response). The AC50 from the Hill is bounded between the minimum log10(concentration) minus 2 and the maximum log10(concentration) plus 0.5. For the gain portion of the gain-loss curve, the AC50 is bounded between the minimum log10(concentration) minus 2 and the maximum log10(concentration). Thus, for both the Hill and gain-loss models, the AC50 is allowed to fall outside of the screened concentration range, with more tolerance for this behavior when it occurs at lower concentrations (or to the left on the x-axis). This is justified from a toxicology perspective because in high-throughput screening, extremely potent chemicals, often used as a reference, may be screened blindly at the same concentration as all other chemicals. As such, these extremely potent chemical may achieve the maximum signal in the assay at the lowest concentration tested, resulting in an AC50 being predicted to fall below the screened concentration range. The slopes for the Hill function and gain portion of the gain-loss function are constrained from 0.3 to 8. These bounds are primarily motivated by the numerical behavior of the fitting algorithm in extreme data configurations. The two extremes are: a) data sets with all non-control responses at about the same level, indicating that all the dose-response must have occurred below the lowest concentration used; and b) the response is elevated above background only at the highest concentration. In the former case, the ML estimate for the power parameter is 0, but the iterative algorithm optimizing the loglikelihood function can become unstable as the estimate of the power parameter approaches 0. For practical purposes, returning a value of 0.3 for that parameter is good enough, and saves time. In the latter case, the ML estimate for the power parameter is infinity. Practically, often the iterative algorithm will terminate at some large value, but again, numerical instability sometimes appears, resulting in an exception that must be handled. The value 8 is a reasonable upper bound for our purposes.

Including a parameter for variance, the cnst, hill, and gnls models have 1, 4, and 6 parameters, respectively. The winning model is determined by choosing the model with the lowest Akaike information criteria (AIC) [70]. For each assay, a value for a cutoff is chosen, either based on the bmad or a value selected. If the winning model is the hill or gnls model, the *tp* parameter is greater than the cutoff, and for at least one concentration the median response value is greater than the cutoff, the curve is declared a hit and parameter *hitc* is set to 1. If one of these three criteria is not met, the curve is not a hit and *hitc* is set to 0.

### Bootstrap uncertainty quantification

The approach used in this study to estimate the uncertainty in model parameters is smooth nonparametric bootstrap resampling:

1. Given *N* concentration-response measurements at *n* concentrations with *j(i)* response measurements at the *i*^*th*^ concentration, *X* = (*x*_1_, *y*_1_),…,(*x*_*n*_, *y*_*j*(*n*)_), sample *j(i)* times at the *i*^*th*^ concentration with replacement *X*_*i*_ = (*x*_*i*_, *y*_*i*,1_), (*x*_*i*_, *y*_*i*,2_),…,(*x*_*i*_, y_*i*,*j*(*i*)_) at all *i*, generating *N* resampled concentration-response measurements $X_{i}^{*}=(x_{i},y_{i,a}),(x_{i},y_{i,b}),\ldots ,(x_{i},y_{i,c})$.
2. Then, to the resampled values $X_{i}^{*}=(x_{i},y_{i,a}),(x_{i},y_{i,b}),\ldots ,(x_{i},y_{i,c})$ add random normally distributed noise (mean zero, standard deviation equal to the bmad) to each value.
3. The resulting values $X_{i}^{*}=(x_{i},y_{i,a}+\upsilon_{1}),(x_{i},y_{i,b}+\upsilon_{2}),\ldots ,(x_{i},y_{i,c}+\upsilon_{j}[i])$ are combined for all *i* to give a resampled set of concentration-response values with the same number of response values as the experimentally measured data.
4. The resampled curve X* is fit to the three ToxCast models to generate point estimates, a winning model, and a hit call.
5. This procedure is repeated 1000 times.

**Fig 7:**
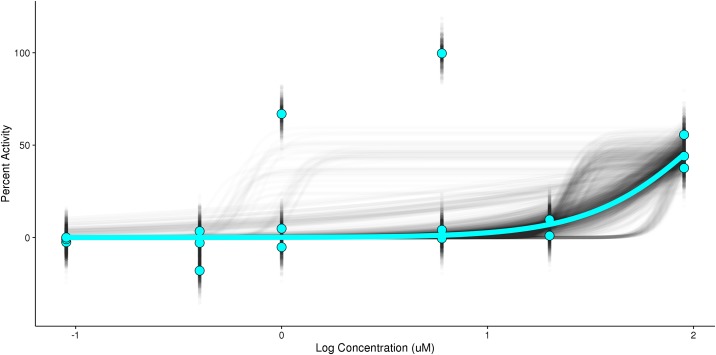
Smooth bootstrap resampling. Normalized experimental concentration-response points (cyan circles) and corresponding hill model (cyan line) are shown. The distribution of smooth bootstrap resampled points (black circles) and fitted values (black lines) are indicated, highlighting the range of resampled observations for response values and the subsequent possibilities for the fitted hill model.

Fig 7 illustrates the sampled points and sampled fits relative to experimental concentration-response values and the curve fit to experimental points. A video showing individual bootstrap resamples and the corresponding fit is included in S1 Video, 10.23645/epacomptox.6062650.

### Implementation

All calculations were performed using R version 3.2.3 (2015-12-10) [71]. An R package toxboot version 0.1.0 [72] was developed to perform all bootstrap resampling. This package makes use of the ToxCast Data Pipeline R package tpcl version 1.2.2 [68] to retrieve the pipeline normalized data and fit the models. Calculations on bootstrap resampling results were made possible using R package data.table version 1.9.6 [73]

### ToxCast ER model for bioactivity

The ToxCast ER model for bioactivity has been described previously [37,38,62]. Briefly, for each chemical the computational model integrates the ToxCast pipeline results from all 18 ER assays. At each concentration, the calculated response from the ToxCast winning model from all assays are summed linearly such that each assay contributes equally to the score. For curves fit to the gnls model, only the gain component was used to calculate the response. Assays where the chemical is not a hit do not contribute to the score. At each concentration, a linear-model is fit to minimize the difference between the measured and predicted activities. For each chemical, the model fits 26 AUC values corresponding to the Agonist, Antagonist, pseudoreceptor or single-assay pseudoreceptor modes. This model was calculated for all 1811 chemicals common in the 18 assays found in the October 2015 ToxCast invitrodb_v2 release.

To calculate the uncertainty in the ER activity scores, the bootstrap resampling values were propagated through the ER model. For a given chemical, the bootstrap results for each assay were indexed 1 to 1000. The matching index values from the 18 assays were paired and the corresponding model parameters were used as inputs for the model exactly as the pipeline values were used to calculate the initial point estimates. This procedure generated 1000 values for each chemical/receptor pair. Subsequent analysis uses the point estimate found from the ToxCast pipeline values with 95% confidence intervals calculated from the bootstrap results by calculating the 2.5% and 97.5% quantile from the distribution of bootstrapped ER model score values.
